# Supplementary figures and images for: Revealing and reshaping attractor dynamics in large networks of cortical neurons
Source: PLoS Comput Biol. 2024 Jan 19;20(1):e1011784. doi: 10.1371/journal.pcbi.1011784 (PMC10829997; doi:10.1371/journal.pcbi.1011784)

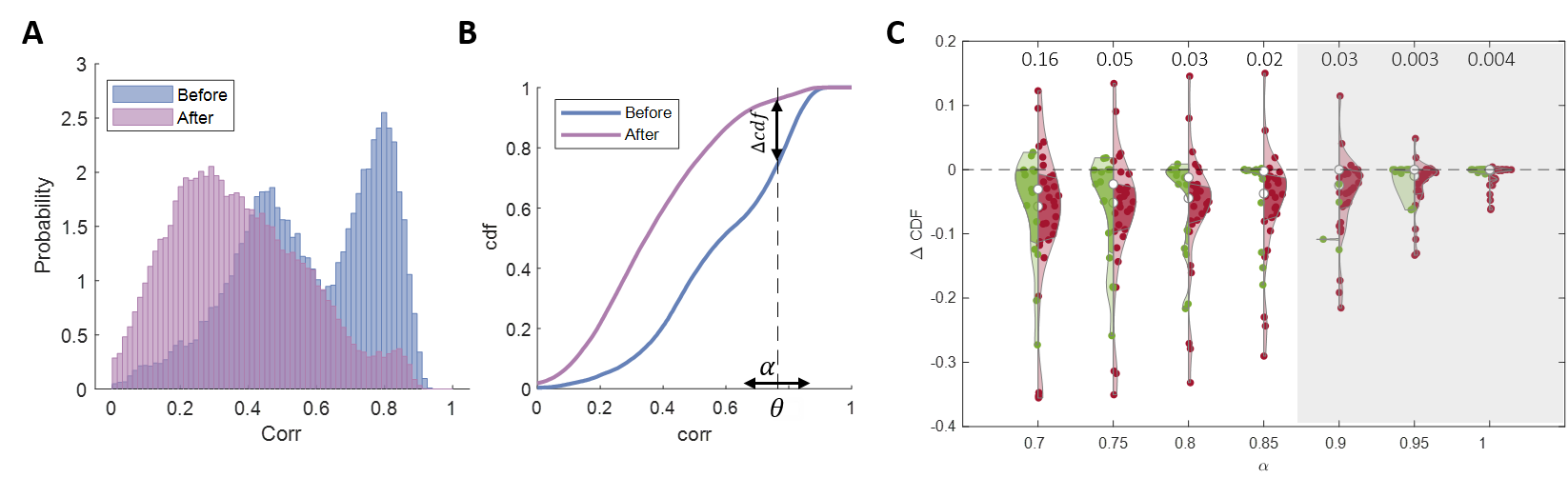

Supplement: S1 Fig — (TIF) [file pcbi.1011784.s001.tif]

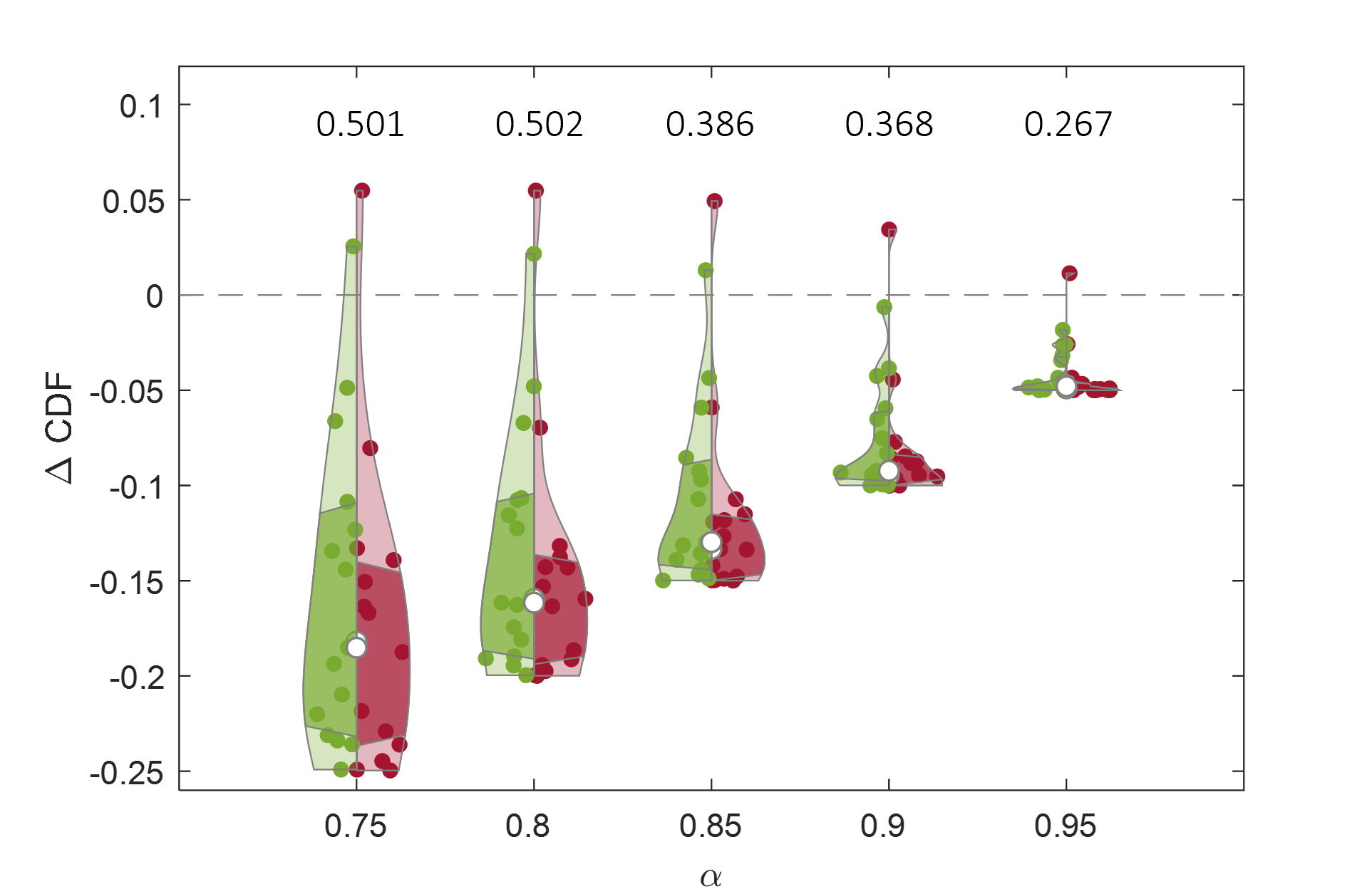

Supplement: S2 Fig — (TIF) [file pcbi.1011784.s002.tif]

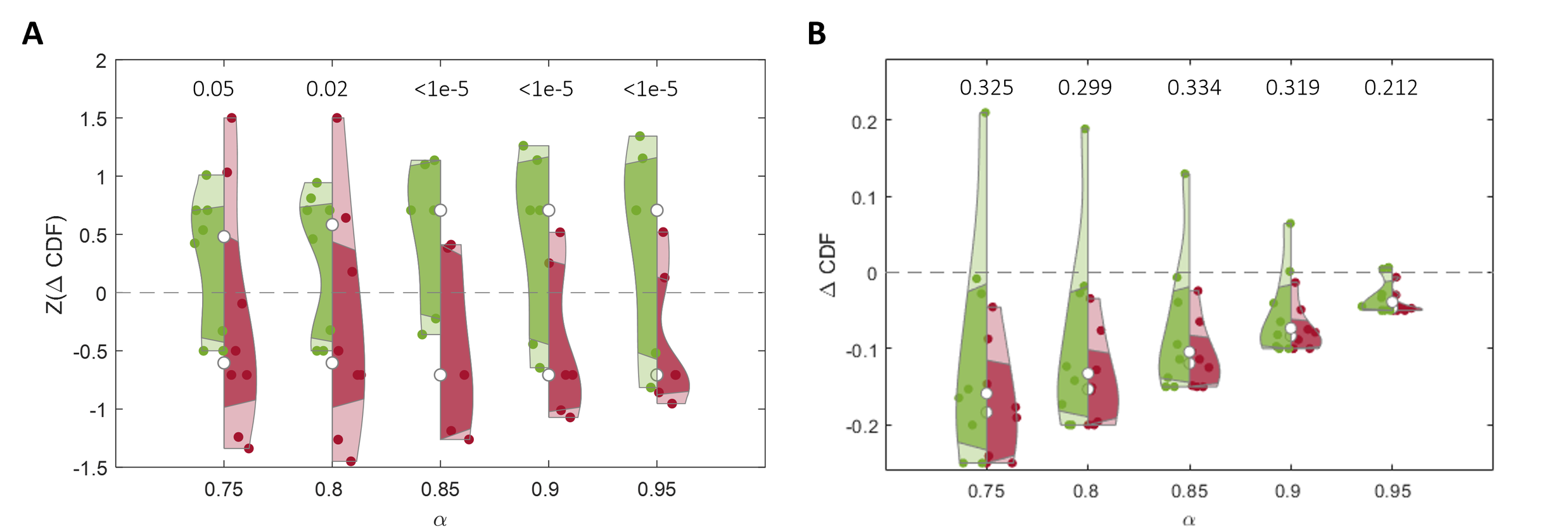

Supplement: S3 Fig — (TIF) [file pcbi.1011784.s003.tif]

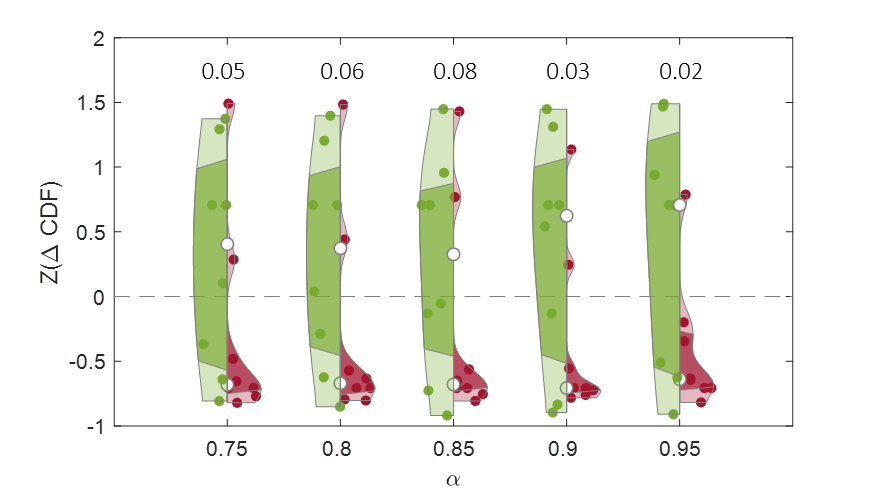

Supplement: S4 Fig — (TIF) [file pcbi.1011784.s004.tif]
