# Supplementary material for: Revealing and reshaping attractor dynamics in large networks of cortical neurons
Source: PLoS Comput Biol. 2024 Jan 19;20(1):e1011784. doi: 10.1371/journal.pcbi.1011784 (PMC10829997; doi:10.1371/journal.pcbi.1011784)
Supplement: S1 Text — (PDF) [file pcbi.1011784.s005.pdf]

Supplemental Material

Measuring the effect using the probe evoked responses

An alternative way to quantify the changes in the spontaneous vocabulary is by measuring the existence of the *evoked responses* in the spontaneous vocabulary of the network. For instance, we can correlate the evoked responses to site 17 (Fig 3E in the main text) to all 1017 spontaneous bursts that occurred before stimulation. The histogram in Fig 1A (blue) shows a large peak in high correlation values, consistent with the fact that this pattern is part of the vocabulary. Repeating the same analysis, this time comparing to the 2092 spontaneous bursts from the period after the stimulation, results in a very different distribution (Fig 1A, purple). These results are similar to the ones of cluster 5 in the main text.

Here as well, we quantify the changes in the existence of patterns using the cumulative probability distribution, exemplified in Fig 1B for the two distributions mentioned above. This time, we use the following alternative metric to measure the effect:

The actual correlation values differ between networks, which is why we use a network-specific threshold as a reference (θ, See Fig 2A in the main text). Using this threshold, we can calculate the change in the existence of high-correlation patterns – $\Delta CDF(\theta)$ (Fig 1B). Here, the difference between control and stimulation experiments is statistically significant.

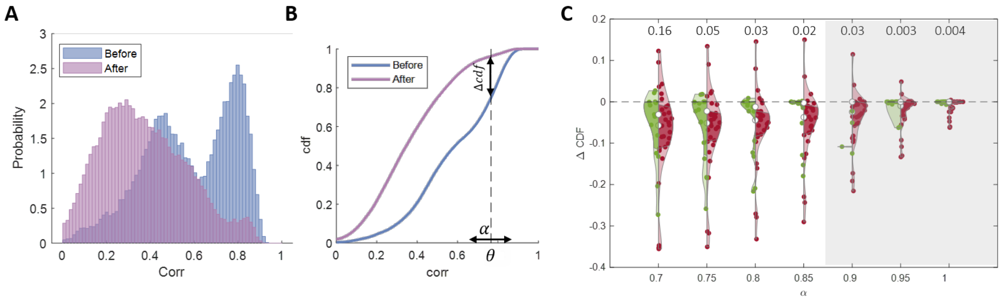

Fig 1. Changes in spontaneous activity – based on evoked responses. (A) 2D correlation values between all spontaneous bursts and the evoked responses to site 17, before (blue, high correlations) and after (purple, low correlations). **(B)** CDFs of the 2 distributions shown in (A). We quantify the effect by calculating the difference at $\alpha\theta$, were θ is the similarity threshold of the network and α is a value in the range [0.71.1]. **(C)** Existence of effect – stimulation vs. control. Statistics across 11 stimulation experiments (Table 1 in the main text) and 5 control experiments (Table 2 in the main text). The violins represent $\Delta CDF(\alpha\theta)$ values in stimulation experiments (red) and in control experiments (green) for a range of α values (the fraction of θ at which ΔCDF was calculated). The numbers above each pair of violins represent the p-value of the hypothesis that the effect in the stimulation experiments is larger than in the control experiments. In the grey area ΔCDF was zero for some of the data points (the CDFs reached 1 for both before and after).

Specificity of the effect – raw values

As explained in the main text, networks differ in their baseline drift rates. We therefore z-scored the ΔCDF values within each network before combining them across networks (Fig 6D in the main text). Fig 2 shows the raw ΔCDF values. We can see that the overall trend still exists, but without statistical significance.

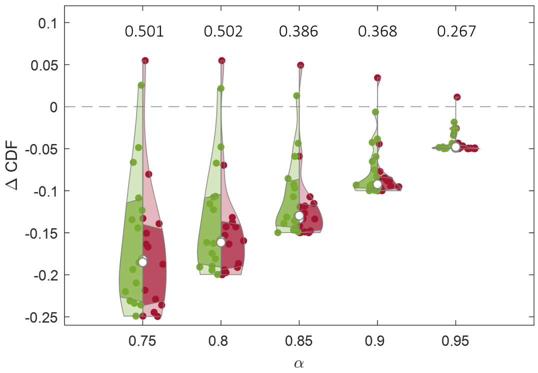

Fig 2. Specificity: raw values. The violins represent the ΔCDF values for the stimulated clusters (red) and for the non-stimulated clusters (green). The numbers above each pair of violins represent the p-value of the hypothesis that the effect in the stimulated clusters is larger than in the non-stimulated clusters.

Specificity of the effect – control experiments

The specificity of the effect in the main text was based on the stimulation experiments (Fig 6D in the main text). In Fig 3 we repeat the same analysis, this time with the 5 control experiments. We can see that the same trend still holds, but the effect is weaker (compare the red distributions in Fig 6D in the main text). This probably means that the robust patterns have a higher drift than the patterns used as controls.

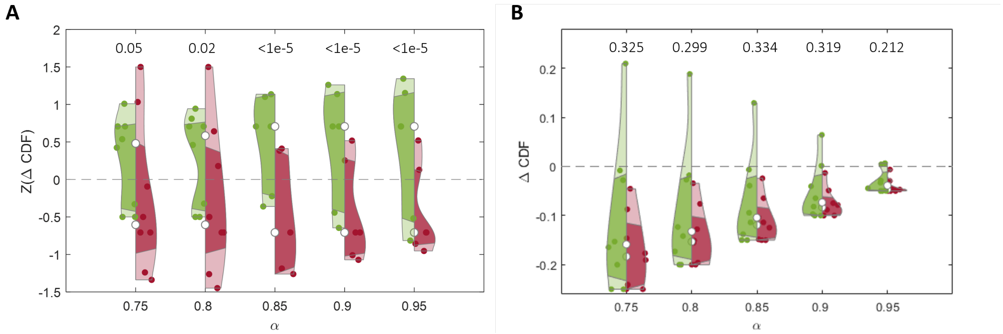

Fig 3. Specificity: control experiments. The violins represent the ΔCDF values for the stimulated clusters (red) and for the non-stimulated clusters (green). The numbers above each pair of violins represent the p-value of the hypothesis that the effect in the stimulated clusters is larger than in the non-stimulated clusters. **(A)** Z-scored ΔCDF values. **(B)** Raw ΔCDF values.

Mechanism – control experiments

The mechanistic explanation for the effect in the main text was based on the stimulation experiments (Fig 8B in the main text). In Fig 4 we repeat the same

analysis, this time with the 5 control experiments. We can see that the same trend still holds, but the effect is weaker.

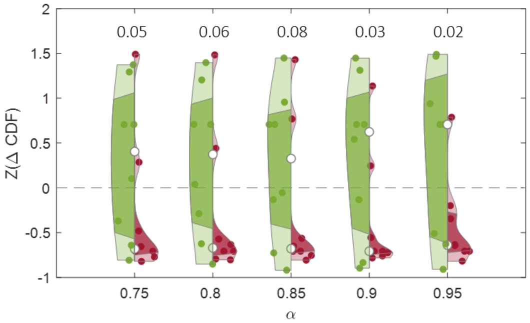

Fig 4. Mechanism: control experiments. Statistics across 5 control experiments. The violins represent ΔCDF values in stimulation clusters (red) and in non-stimulated clusters (green) for a range of α values. The numbers above each pair of violins represent the p-value of the hypothesis that the effect in the stimulation clusters is larger than in the non-stimulated clusters.
