# Supplementary material for: Revealing and reshaping attractor dynamics in large networks of cortical neurons
Source: PLoS Comput Biol. 2024 Jan 19;20(1):e1011784. doi: 10.1371/journal.pcbi.1011784 (PMC10829997; doi:10.1371/journal.pcbi.1011784)
Supplement: Attachment — Submitted filename: Response.pdf [file pcbi.1011784.s006.pdf]

TECHNION
Israel Institute
of Technology

Omri Barak
Associate Professor

Rappaport Faculty of Medicine, and
Network Biology Research
Laboratories
P.O.B. 9649, Bat-Galim, Haifa 31096
Israel

Tel. +972-4-8295322
omri.barak@gmail.com
barak.net.technion.ac.il

Revised manuscript: Revealing and reshaping attractor dynamics in large networks of cortical neurons

Dear Editors,

We appreciate your consideration of our manuscript, "Revealing and reshaping attractor dynamics in large networks of cortical neurons," and we thank the editorial board and the three reviewers for their valuable feedback.

All reviewers expressed concerns on the clarity and terseness of the manuscript, and we took care to be much more clear and elaborate in the revised version.

In addition, we have carefully considered each comment. Below, we outline our responses to the reviewers' comments and provide a brief description of the changes made in the revised manuscript.

Sincerely,

Chen Beer and Omri Barak

Major changes:

- We simplified the evaluation metric for measuring changes in spontaneous activity (new definition of α in Figures 6,8). The previous metric is in supplement Fig 1.
- We added a supplemental material section with additional analyses, inspired by the reviewers
- We clarified and extended our explanations throughout the main text and in the methods section, including new illustrating figures
- We improved all figures and their captions
- We added 9 references

✓ We uploaded all our data and code, and they are now available here
✓ All figures were uploaded and approved by PACE

Detailed responses to reviewers' comments:

Reviewer #1

Presentation:

1. In Figure 2, you should show to the reader the correspondence between the attractors plotted in panel C and those in the embedding in panel D. There are nine attractors and nine trajectories in the embedding. I can probably guess which ones correspond between C and D, but you could also label them to help the reader.

We added the cluster number labels next to each of the median trajectories in the UMAP space. Note that the UMAP space does not have a simple correspondence to the physical one presented in panel C. Please refer to the updated figure below, which has replaced the previous one:

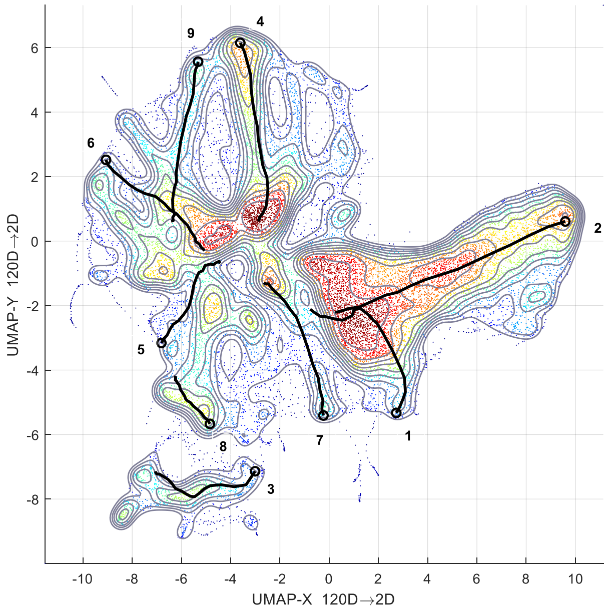

Regarding panel D, I looked for a citation or brief explanation of UMAP but could not find one. While the paper is very widely cited, you should still give readers some indication of what you are doing.
Thank you for bringing this to our attention, the UMAP paper is now cited in the manuscript.

No colorbar is shown for figure 2D, only contour plots without explanation. Again, while I can probably guess or work it out, you could also help the reader here.
We have now included an explanation in the figure's caption to clarify the meaning of the contour plots.

In panel C, are the circles the beginnings of the trajectories? I did not see that indicated until much later in the caption for figure 8, panel C.
Yes, the circles in panel C do represent the starting points of the trajectories, as stated in the caption of Figure 2C.

2. Reference 10 seems like a non-sequitur.
Thank you, now fixed.

3. In the methods section, under Data processing, you say that you bin the data in bins of 5 ms. Do you ever get more than one spike in a single bin? If so, how do you handle it? It could be that you just give a 1 if there are two spikes there, or you could give a 2, counting both spikes. How often did you get multi-unit activity (MUA)? How often did you get clear single spikes? Were there any local field potentials that could have produced threshold crossings? If so, did you discard these or count them as activity at the electrodes? I could not find where these issues were addressed.
All these important points are now clarified in the data processing part of the methods sections.
We do not sort the spikes, and therefore only consider MUA.
To avoid counting LFP as threshold crossings, we ensured clear spike events by starting recordings only after the culture had rested on the MEA for at least 30 minutes and setting a conservative threshold of 5 standard deviations. We visually confirmed that threshold crossings only corresponded to clear spike events and nothing else. Only then was the actual recording started.
Binning was done after gaussian smoothing, so the actual value in a given bin can be both lower than one and higher than one. A new figure in the Methods section clarifies this now.

4. Under Clustering Method, your explanations are unclear to me. For example, in describing the spatiotemporal correlation, are you performing vector subtraction element by element in the numerator of the equation? That is my guess, but you could be clearer. In describing the Correlation of spatial profiles, are you taking the amplitude to be the sum of the active

electrodes at each time bin? Please explain. Perhaps a toy example illustrating your methods could help here.

To enhance clarity, we have extended this section in the Methods and included an explanatory figure.

In the equations provided, $\bar{A}$ and $\bar{B}$ represent averages over both dimensions (electrodes and time). This is essentially equivalent to flattening both A and B matrices and then calculating the Pearson correlation. We have expanded and improved the explanation in the Methods section to make this clearer.

Regarding the Correlation of spatial profiles, you are correct. We sum the activity of each electrode across all time bins and then calculate the correlation between the resulting two vectors of length N.

5. On line 86, you mention that you use spectral clustering methods to group the trajectories with the most similarity into your vocabulary. While I have used spectral clustering methods, I think you should describe to the reader a bit more how you did this. Did you use a greedy algorithm, or did you anneal for your solutions? This is a computational biology journal, so I think you should describe a bit more about the steps in your computations.

We now explain this process in detail in the Methods section, lines 374-385.

Briefly, we start from the graph described in the methods (nodes are bursts, and weights have a value of 0-3). We then compute the normalized symmetric graph Laplacian, and use k-means clustering on a normalized matrix derived from the first k eigenvectors. We used a greedy algorithm via the spectralcluster function.

6. I found Figure 9D to be a challenge. You offer only a brief explanation of what is presented there, and I cannot see the axes. Are there directed connections there or are all the connections undirected? Why does the plot seem to look like it has a void in the middle? Does this tell us anything? What important point of your paper is it supporting? This was not obvious to me.

The aim of the figure was to show how the graph leads to clustering of bursts. First, it was indeed poorly explained, and a complete explanation is below. Second, we decided that it does not contribute much and removed it from the revised version. If you believe it would be helpful, we can include it in the supplement.

Clustering begins by forming an abstract graph in which each node is a single burst, and each edge has a weight from zero to three, signifying the number of similarity metrics that passed the threshold for the specific pair of bursts. The new version of the figure below shows an embedding of this graph on the plane, with the shading of edges denoting their strength. One can see several clusters of nodes, corresponding to the clusters of bursts detected by spectral clustering applied on this graph. The exact shape is not informative, as it is the result of embedding a high-dimensional graph in 2D for visualization purposes.

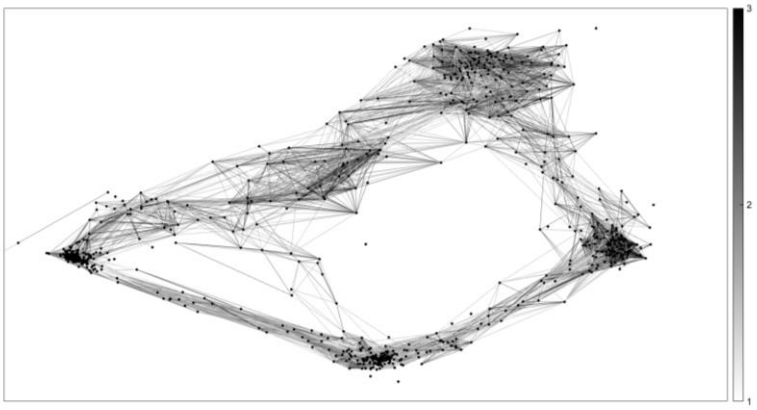

7. In some of the figures, you use COM to presumably describe Center of Mass. Please just spell it out in the captions so people do not have to guess what it means.

It is now written explicitly.

Scientific issues:

1. One of my main concerns is over whether you actually have attractors or not. I think I get the logic behind figure 2 B, where the variance declines over time. I really like that, but I am wondering about what happens if you plot this longer than 0.15 seconds. Does the variance go up again after that? Why stop at 150 ms? I would like to see if they are becoming more similar over time merely because more electrodes are getting activated. If most of the array is just becoming activated, then of course nearly any starting configuration will look more similar over time. There would be just one large attractor like what you might see in a seizure. Please convince me that this is not the case.

This is a great question, thank you for raising this concern.

Briefly, after 150msec, activity usually goes down to baseline, as opposed to all electrodes becoming activated.

Below is an extended version of Figure 2B for a longer duration of 0.25 seconds, limiting the analysis to bursts lasting at least 0.25 seconds (Now Figure 11 in Methods). In this extended plot below, you can observe that the variance does indeed increase after approximately 0.15 seconds. In addition, we plotted the average overall activity (sum over all electrodes) through time. These additional visualizations provide evidence that the attractors are not simply a result of the entire array becoming activated. We hope this clarification addresses your concern.

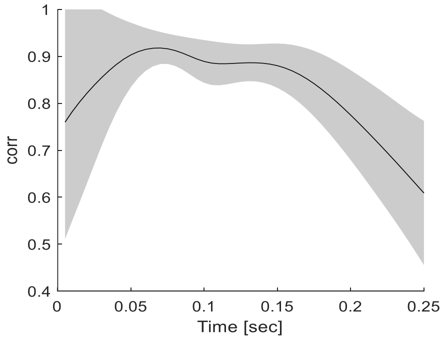

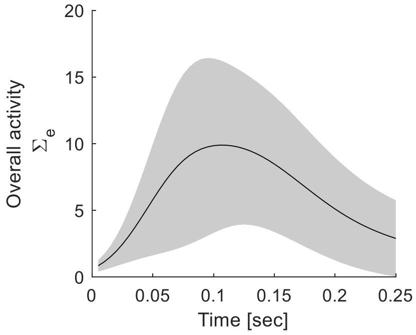

2. Any time a threshold is chosen, one may wonder how sensitively the results depend on its exact value. If you take the threshold theta for clustering correlations described in the caption to figure 2A and change it by say + or – 20%, do you still get qualitatively the same results? This would give me some more confidence if you could show it.

Indeed this is an important concern and the reason for the alpha parameter in figures 6,8.

Clustering is used in the paper for two purposes: to show the structure of spontaneous activity, and to show the reduction in frequency of stimulated attractors. Both conclusions are also supported by alternative analyses.

For the structure, the UMAP visualization shows the same structure without relying on clustering.

For the change in spontaneous activity, we performed an alternative analysis (Figure 15 in supplementary) that relies on **the probe evoked responses instead**. Briefly, we compare all *spontaneous* bursts to the *evoked* bursts. This result is statistically significant, and does not rely on the clusters at all. The reason we present in the main text the analysis based on the spontaneous clusters is due to the intricacies involved in comparing evoked responses to spontaneous bursts (excluding 6 stimulated electrodes for each comparison).

In response to the reviewer comment, we also conducted clustering analyses with thresholds set to both +10% and -10% with respect to the threshold described in the caption of Figure 2A (+20% was above 1). We applied this sensitivity analysis to the same culture presented in the paper. The results remained qualitatively the same under these variations – see below.

The threshold primarily determines the cutoff for each cluster. For larger thresholds, each cluster contains fewer bursts, leading to a lower overall percentage of clustered events. However, these variations do not alter any of our main conclusions.

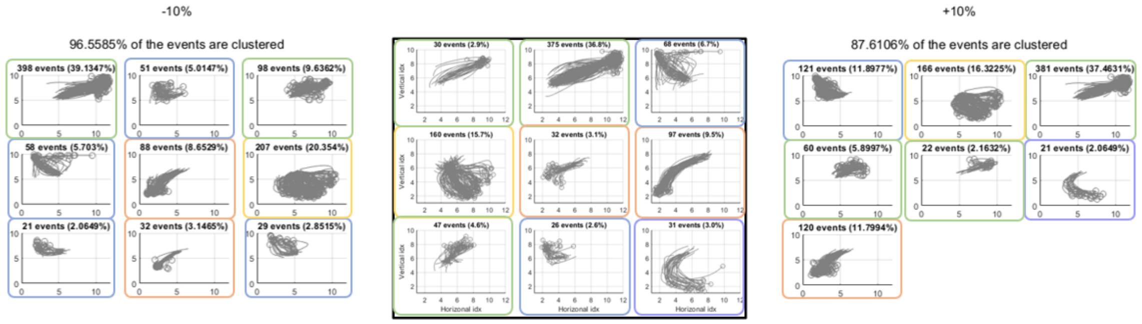

3. When discussing the artifacts caused by electrical stimulation (line 120), you did not mention doing a TTX subtraction. As you probably know, washing in TTX will show you what portion of the artifact was caused by capacitance and resistive effects that do not depend on sodium channels. This signal can later be subtracted from the full recorded waveform to reveal only that portion that was caused by neuronal firing. Of course, you only want to wash in the TTX at the very end of the experiment when you are no longer recording activity. TTX can be washed out, but it takes some time.

We appreciate your suggestion regarding the use of TTX to identify and subtract artifacts caused by electrical stimulation. However, we were not aware of this specific procedure during

our experiment. To ensure that our analysis was not influenced by stimulation artifacts, we took the following measures:

1. We excluded all stimulating electrodes from our analysis when examining evoked responses.
2. We omitted the first 5 milliseconds following stimulation offset from our analysis.

By implementing these precautions, we aimed to minimize the impact of stimulation artifacts.

4. A suggestion on the interpretation of your results: I can see how Hebbian plasticity could account for the fact that the frequently stimulated attractors become more responsive to stimulation over time. But the observation that they are less often spontaneously evoked could be explained by homeostasis. I do not think you mentioned homeostasis, but you should probably consider it. See, for example (Liu, Seay and Buonomano, 2023).

Thank you for this comment. We fully agree that homeostasis is a likely mechanism and accidentally omitted it in the previous version of the manuscript. It is now part of the discussion in the current version of the paper.

Regarding the Liu et al paper, they mention homeostasis at the single cell excitability level. We think of homeostasis at the network level. Specifically, our hypothesis is that Hebbian plasticity strengthens the evoked pathways into a given attractor. At the same time, homeostatic plasticity weakens the spontaneous (non-evoked) pathways into the very same attractor.

Reviewer #2

My only major comments, it is not clear what is new in this study. The structure of synchronized bursting events was analyzed by Ben-Jacob et al, and the effect of stimulus on the network dynamics was demonstrated by Maron et al. It would be nice if the authors explain explicitly what is novel here. In addition, the authors can do a better job in citing prior art in the analysis of synchronized bursting events.

We assume the reviewer is referring to Segev & Ben Jacob, Physica A 2001 and to Shahaf & Marom, JNS 2001.

Regarding the structure of synchronized bursts – we believe the demonstration of attractor dynamics is a novel contribution. This refers both to the convergence of spontaneous events, and to the similarity between evoked and spontaneous.

Regarding plasticity – Shahaf et al did not consider the structure of bursts, nor attractor dynamics. We believe the present work offers a coherent framework for how stimulation interacts with existing dynamical objects to modify them.

We also refer to the evaluation of novelty by reviewer #1 that mentions these and other aspects.

Regarding the citation of prior work, we agree and have improved this along with improving the overall clarity of the manuscript.

Minor comments:

Figure 1A: maybe scale bar missing?

The scale was specified in the methods, it is now written in the figure's caption as well.

Figure 1B right panel, what are axis labels?

The axes represent the electrodes grid, which is organized in a 10x12 configuration. We have updated the figure to include explicit axis labels to improve clarity.

Figure 2 caption: what is upside down '?'?, panel C, what are the axis?

The upside down "?" is now changed to ">".

The axes represent the electrodes grid, it is now written explicitly in the labels.

Figures 3, 7, 8, all panels should have labels and units.

Thank you, all panels in these figures now include appropriate labels and units

Figure 9D: I do not understand panel D.

Please refer to the response provided to Reviewer #1, question number 6 under the "presentation" section. This response includes additional information and an updated version of Figure 9D for clarification.

Reviewer #3

1) In Fig. 2A, we see the density plot for correlation of overall burst vs initial condition. It can be observed a large fraction of burst pairs have close to zero initial correlation but large overall burst correlation. Any ideas where that comes from?

Thanks for the attention to detail that allows us to better explain this aspect.

The typical correlation value between bursts belonging to *different* clusters varies between cultures, as illustrated in the figure below. For the culture presented in Figure 2A, this was indeed around 0.6. This is because all bursts have some things in common – a coordinated increase in activity of multiple electrodes, occurring on roughly the same timescale.

It is important to note, however, that the bimodal distribution of correlation values appeared in all networks, regardless of the correlation baseline. It is this bimodal nature that allowed us to perform clustering of burst events.

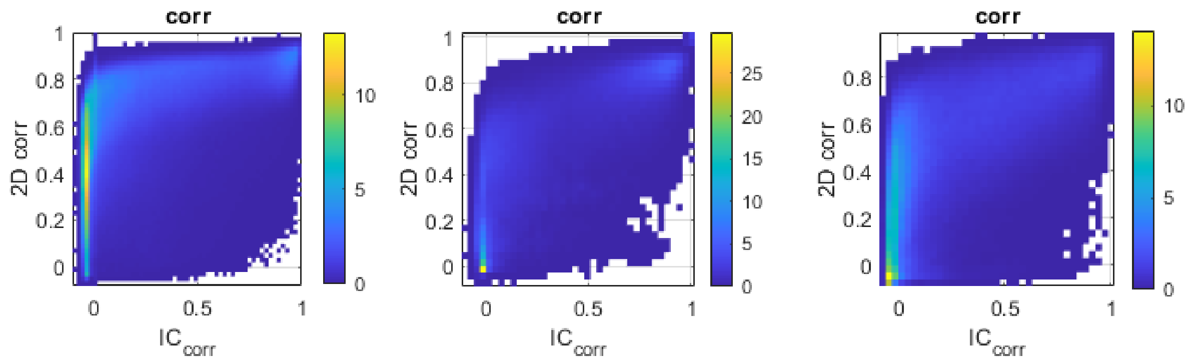

2) In Fig. 2D, we see a low-dimensional representation of the trajectories for all clusters extracted from a single (?) culture. There it becomes clear that all bursts tend to move towards the middle of the MEA. Is there a reason for this? Perhaps a bias on connection densities? It also can be noted that bursts tend to not start at the center, do we know why that is the case?

We apologize for not clearly explaining this figure in the original submission.

First, this is indeed a single culture. The same one used in all of Figure 2.

Second, the UMAP 2D space used in Figure 2D is an arbitrary embedding and is distinct from the physical 2D arrangement of the MEA. The observed tendency of bursts to move toward the center of the UMAP space is primarily due to the fact that the center of the UMAP space corresponds to the origin (0,0), and the activity of the bursts diminishes as they progress towards their end. These aspects are now better explained in the text.

3) Stimulation occurred in a predetermined sequence that is also mostly spatially contiguous (site 1 is close to site 2, which is close to site 3, etc.). Could that have consequences in the responses observed? For instance, if by stimulating site 1, certain neurons become depleted and can't participate when site 2 is stimulated next, that could narrow down possible trajectories in response to site 2 stimulation.

We apologize for not clearly explaining the terminology in the original submission.

The word "site" in Sites 1,2,3 of Figure 5 and the description of the stimulation protocol does not correspond to the site numbers in Figure 3. The chosen stimulation sites (1,2,3) were in fact strategically picked to minimize potential consequences related to spatial contiguity and depletion effects. In the example given in the manuscript the 3 stimulation sites selected for the 10-hour stimulation are sites 3, 16, 17 as illustrated below in green, blue, and red:

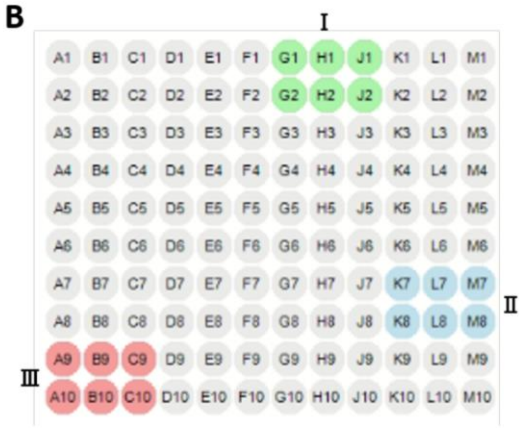

This figure is now included in Fig 5B in the manuscript. In addition, we have changed the notation such that the 3 selected stimulation sites are now denoted in roman letters.

Furthermore, stimulation happened at an interval of 10 s. It seems bursting in spontaneous activity occurred at an average interval of ~14 s (based on numbers given by the authors). Could the shorter interval for stimulation also be a factor here? Maybe the data obtained from the repeated stimulation of specific sites can be used to answer this question.

Regarding the stimulation interval of 10 seconds, it's important to note that the bursting rates before stimulation, as presented in Table 1, range from 5.2 to 13.8 seconds, depending on the specific culture. The evoked responses occurring every 10 seconds are robust in most cases, even considering the maximum spontaneous bursting interval of 13.8 seconds (as seen in the figure provided in response to question 4 below).
It's important to distinguish between the spontaneous bursting rate and the refractory period, which tends to be much shorter. Namely, we can reliably evoke a response at a time that is shorter than the typical spontaneous emergence of a burst. This combination of factors, including site selection and stimulation interval, was designed to ensure the reliability of our experimental results.

4) In Fig. 3B, we see robust and coherent responses to stimulation site 17, but it's intriguing that the initial point in those trajectories is so far removed from the stimulation sites? Any ideas why this is the case? Also stimulation site 10 doesn't seem to yield coherent responses. Could it be linked to the fact that this site is close to the center (maybe related to the question in point 2 above)?
In general, there is a connection between stimulation site and the burst onset place. The following figure contains the network's evoked responses to all 20 stimulation sites (including 17 & 10, which are included in the paper). Regarding the center, as evident in the figure below, coherent responses starting at the center do exist for other stimulation sites, such as 3, 7, 11, and 17.

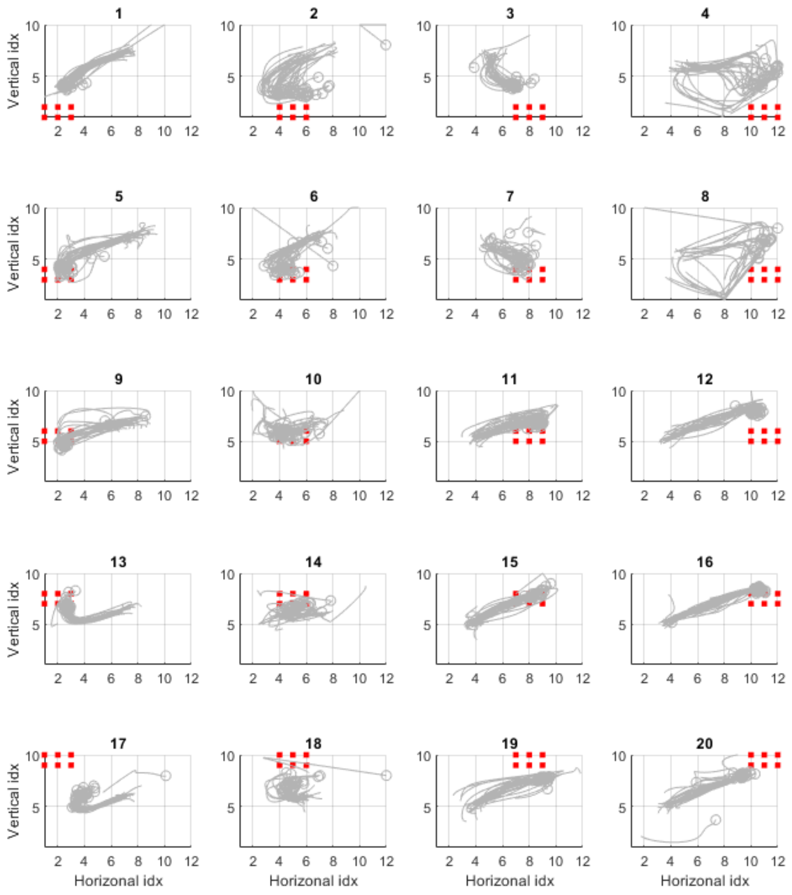

5) In Fig. 4, we see a very small fraction of responses that are very robust (to the right of the vertical dashed line) but couldn't be "explained" by the spontaneous vocabulary. Did the authors further investigate these responses? It would be interesting to understand what conditions give rise to those that are not present during spontaneous activity.

Thank you for this comment.

We inspected the four patterns. Two were found to be spurious results – the firing rate was very low, leading to high correlation without true robustness. We now examined all other patterns, and made sure that none of the patterns used in the later parts suffered from the same problem.

The other two patterns are indeed the only examples in our dataset of robust evoked responses that do not exist in the spontaneous vocabulary. While interesting for further study, we did not pursue it within the scope of this work.

	Identity	Evoked responses	Spontaneous vocabulary	Explanation
1	Control<br>2655024.1<br>site 9	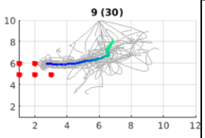	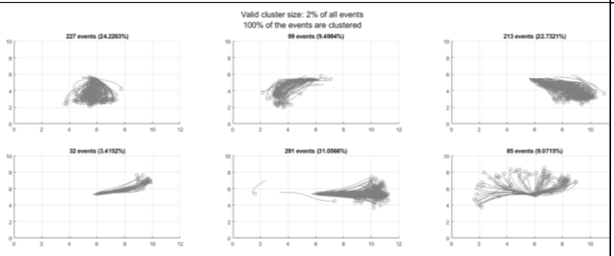	The response is robust, but there is no similar cluster
2	Control<br>265367.2<br>site 9	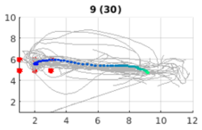	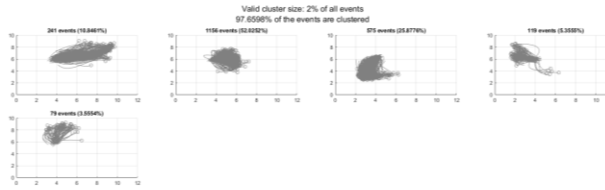	The response is robust, but there is no similar cluster
3	3842820.2<br>site 20	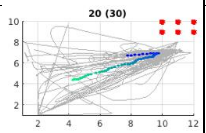	Not relevant	The response is not robust. High correlation is due to very low activity levels.
4	N/A<br>8.11<br>Site 20	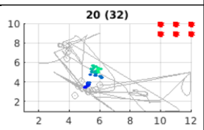	Not relevant	The response is not robust. High correlation is due to very low activity levels.

6) Since the authors do have control experiments in which no stimulation was performed, it would be important to obtain some more statistics on changes to existing clusters after that 10 h period, so the effects of stimulation are taken with more context. For example, one can see in table 2 that the number of clusters change for all cultures. Are these mostly from a few clusters being added/removed from the previous set, or are we seeing completely new set of clusters after 10 h? How similar are the clusters before and after?

We appreciate your suggestion to conduct more detailed statistics on cluster-level changes. However, when we attempted such analyses, we encountered challenges in deriving meaningful insights. It's important to note that the clusters themselves are somewhat arbitrary, as the number of clusters and their sizes in each culture result from various thresholds, as explained in the methods section.

To provide readers with some insight into the changes at the cluster level, we included statistics in Tables 1 and 2, offering a sense of the alterations that occur within clusters. Because of these difficulties, we relied on comparison of single spontaneous bursts to the evoked ones as a means to measure the effects reported in the study.

7) Finally, I would suggest being a little more detailed on the definitions necessary to understand the stimulation part (Figs. 6 to 8). While they can all be found in the Methods section, I believe it would help the reader to follow the results more clearly if they were explained in a little more detail.

We have taken your feedback into account and made efforts to simplify the analyses in Figures 6 to 8 to enhance clarity for the reader. Additionally, we have added a supplementary section that provides more detailed analyses to complete the overall picture. We believe that these adjustments and additions will make this section easier to follow and understand.
